# P-270. Comparing Regression and Machine Learning Methods for Predicting Readmission Risk among People with HIV

**DOI:** 10.1093/ofid/ofaf695.491

**Published:** 2026-01-11

**Authors:** Heather Henderson, Lindsay Browne, Alex Commanday, Amanda E Moy, Claire E Farel, Joseph J Eron, Sonia Napravnik

**Affiliations:** University of North Carolina at Chapel Hill, Chapel Hill, NC; UNC Chapel Hill, Chapel Hill, North Carolina; University of North Carolina at Chapel Hill, Chapel Hill, NC; School of Medicine, University of North Carolina at Chapel Hill, Chapel Hill, North Carolina, USA, Chapel Hill, North Carolina; UNC Chapel Hill, Chapel Hill, North Carolina; University of North Carolina at Chapel Hill School of Medicine, Chapel Hill, North Carolina; UNC Chapel Hill, Chapel Hill, North Carolina

## Abstract

**Background:**

Predictive models can help identify high-risk patients who may benefit from targeted interventions. We evaluated the utility of predictive models for classifying 30-day hospital readmission risk among participants in the UNC CFAR HIV Clinical Cohort during 2014-2024.

**Table 1:**
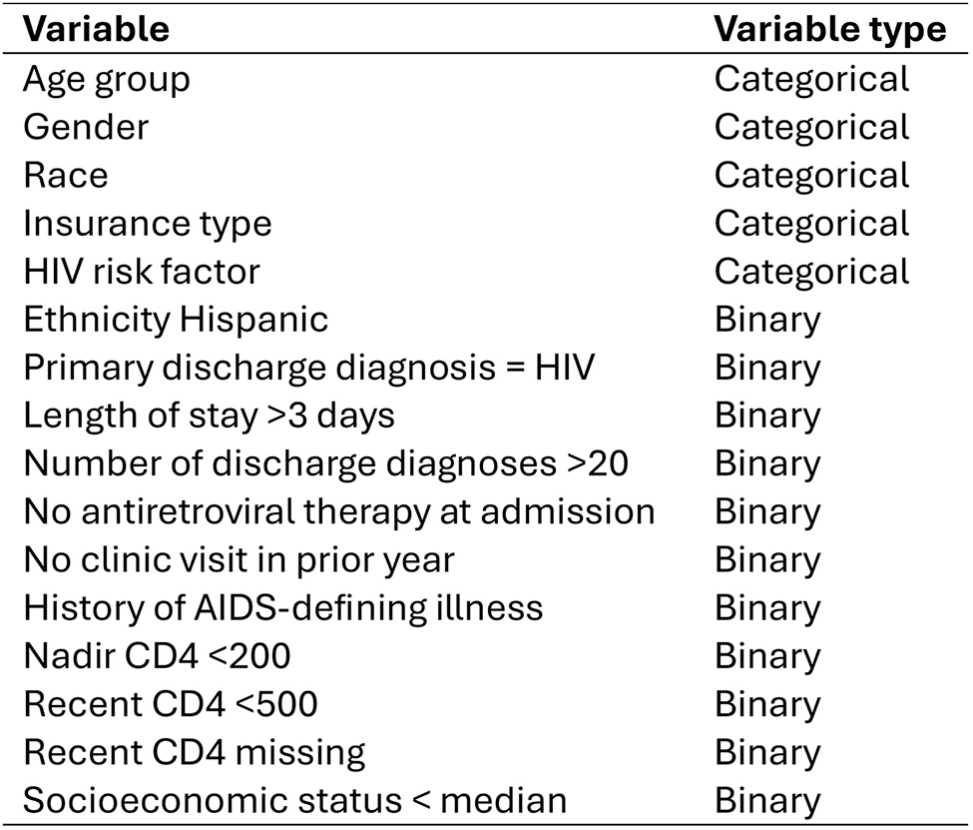
Candidate predictors included in models

**Table 2:**
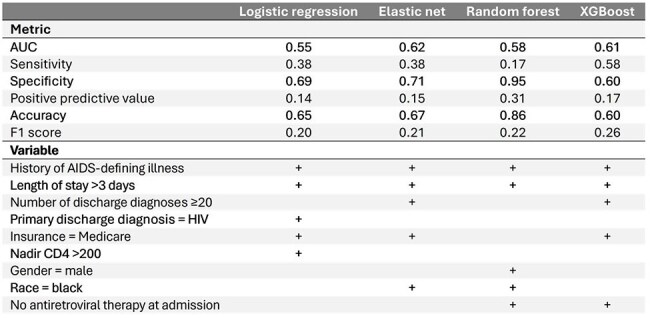
Summary of model evaluation metrics and most influential predictors

**Methods:**

We compared logistic regression (LR), elastic net (EN), random forest (RF) and extreme gradient boosting (XGBoost) models. Readmission was defined as an admission within 30 days after discharge. Models were trained on a 75% random sample of data with 25% held out for testing. All models except LR used 5-fold cross-validation for hyperparameter tuning. We included readily available clinical and demographic predictors (Table 1) and evaluated model performance using metrics including area under the receiver operator curve (AUC), sensitivity and specificity. The variables most influencing predictions were determined using model coefficients (LR, EN) or built-in feature importance (RF, XGBoost). In separate multivariable models we estimated adjusted risk ratios (aRRs) for the top predictors.

**Results:**

Among 804 participants with an index hospitalization, 98 (12%) were readmitted. Predictive performance was limited, with AUCs ranging from 0.55 (LR) to 0.62 (EN) (Table 2). Length of stay > 3 days and history of an AIDS-defining illness (Hx-ADI) were among the top predictors across all models. Primary discharge diagnosis of HIV, nadir CD4 > 200 cells/µl and male gender were each included in only 1 of the 4 models. Length of stay > 3 days and Hx-ADI were each associated with significantly increased readmission risk in multivariable models: aRR 2.4 (95% CI 1.5-3.8) and 1.7 (95% CI 1.1-2.7), respectively.

**Conclusion:**

Predicting 30-day readmission in this population remains challenging. Larger datasets and inclusion of additional predictors, including social and behavioral factors, may be necessary for improved performance. We identified length of stay > 3 days and Hx-ADI as important predictors, each associated with approximately doubled risk of readmission. Although lacking biological plausibility, nadir CD4 > 200 cells/µl was among the top predictors in the LR model. This shows that for a generalizable model, regularization tuned by cross-validation is necessary to minimize the impact of spurious associations.

**Disclosures:**

Joseph J. Eron, MD, Gilead Sciences: Advisor/Consultant|Gilead Sciences: Grant/Research Support|Merck: Advisor/Consultant|Merck: Grant/Research Support|ViiV Healthcare: Advisor/Consultant|ViiV Healthcare: Grant/Research Support

